# Telerehabilitation in India: From awareness to acceptance

**DOI:** 10.6026/973206300211689

**Published:** 2025-06-30

**Authors:** Saurabh Agnihotri, Naveen Kumar Singh, Nalina Gupta

**Affiliations:** 1 Department of Physiotherapy, Teerthanker Mahaveer University, Delhi Road, Moradabad - 244001, Uttar Pradesh, India; 2 Department of Surgery, Teerthanker Mahaveer Medical College, Teerthanker Mahaveer University, Moradabad, India; 3 MM College of Physiotherapy and Rehabilitation, Sadopur, Ambala, Haryana, India

**Keywords:** Telerehabilitation, knowledge-attitude-practice, telemedicine, digital literacy, public health, cross-sectional survey

## Abstract

Telerehabilitation is gaining prominence in improving healthcare access, especially in underserved regions. Therefore, it is of interest to evaluate the knowledge, attitudes and practices (KAP) of the general population in Western Uttar Pradesh, India. A cross-sectional survey of 455 individuals was conducted using a validated, Hindi-translated questionnaire. While only 40.7% were previously aware of telerehabilitation, 81.8% showed willingness to use it, particularly among younger, male and better-educated participants. Thus, the need for targeted awareness and digital inclusion strategies to enable equitable access to telerehabilitation across populations is reported.

## Background:

Telerehabilitation, a branch of rehabilitative medicine enables the remote delivery of physiotherapy services through digital technologies, particularly benefiting patients in underserved regions [1]. It allows personalized treatment for various conditions, including musculoskeletal, neurological, cardiopulmonary and pediatric disorders, via internet-based systems, messaging services and audio/video calls [2]. Telerehabilitation has been shown to improve continuity of care and accessibility, particularly in resource- limited settings [3].

Studies have shown that home-based telerehabilitation provides clinical outcomes comparable to traditional face-to-face therapy, particularly in patients recovering from total knee arthroplasty [4]. It also promotes adherence and reduces overall healthcare costs [5], while eliminating geographical barriers to access [6]. India's national initiatives such as Sanjeevani and MANAS (Mental Health and Normalcy Augmentation System) have successfully integrated telerehabilitation into mainstream healthcare by offering remote consultations and mental health support especially during the COVID- 19 pandemic [7, 8]. Despite these advancements, digital illiteracy infrastructure limitation and cultural hesitation continue to obstruct widespread adoption of telerehabilitation [9]. Therefore it is of interest to report the knowledge, attitude and practice of telerehabilitation among the general population of Western Uttar Pradesh.

## Methods and Materials:

## Study design:

This was a cross-sectional observational study.

## Study population:

The target population included residents of Western Uttar Pradesh, specifically focusing on individuals living in Moradabad and nearby districts. This demographic was chosen to provide insights into the telerehabilitation knowledge, attitude and practice (KAP) within this specific geographic area.

## Source of data:

Data were sourced directly from the general population residing in Moradabad and adjacent districts.

## Sample size:

Based on the assumption that 50% of the target population was knowledgeable about telerehabilitation, the sample size was calculated at 454 individuals using formula.

Z^2^ pq/d^2^

Where z is taken to be 1.96 for 95% confidence limits with p = 50% (anticipated knowledge level), q = 100 - p and d denoted the absolute error in precision which was 5%

## Inclusion criteria:

[1] Age: Participants above 18 years.

[2] Residency: Residents of Moradabad and surrounding districts in Western Uttar Pradesh.

[3] Gender: Both male and female participated in the study.

## Exclusion criteria:

[1] Participants unwilling to participate.

[2] Participants unable to comprehend the questionnaire.

## Procedure:

The study began with the development of a questionnaire, initially comprising 10 questions aimed at assessing the KAP of telerehabilitation among the general population in Western Uttar Pradesh.

## Questionnaire development:

The development process of this self-designed questionnaire included multiple stages to ensure it was comprehensive, reliable and culturally relevant to the target population. The initial design consisted of 10 questions, distributed across three domains Knowledge, Attitude and Practice each aimed at capturing distinct aspects of the population's interaction with telerehabilitation. Reliability was confirmed by pilot testing (Cronbach's alpha=0.78). Translated into Hindi for cultural appropriateness, it was pre-tested on 20 participants.

## Ethical considerations:

The study was approved by the Institutional Ethics Committee, College of paramedical Sciences, Teerthanker Mahaveer University (Approval No.: PM/ETHICAL/PT/2023/004).

## Data collection:

Data collection was carried out by trained physical therapists who had also participated in the pre-testing phase of the study. The process took place in November and December 2023 in Bagadpur and other areas of Moradabad. Participants were approached at their homes or workplaces, where written informed consent was obtained prior to data collection. They were informed of their rights, including the option to withdraw from the study at any point during the interview. After completing the questionnaire, participants received information about the telerehabilitation services provided by the Physiotherapy Department of Teerthanker Mahaveer Hospital.

## Data analysis:

Data analysis was done using Strata 16.0 software.

## Results and Discussion:

A total of 455 individuals participated in the study. The majority (62.7%) were aged 18-30 years, followed by 34.0% in the 31-60 age group and 3.3% above 60 years. Males comprised 65.4% of the sample, while females represented 34.6%. In terms of education, 39.1% were graduates, 26.3% had completed senior secondary education and 5.7% were uneducated. Table 1 (see PDF) presents the demographic distribution). Only 40.7% of participants were previously aware of telerehabilitation; however 81.8% preferred it for consultations. Most found it easy to use (70.5%), cost and time efficient (74.2%) and accessible (78.9%). Furthermore, 84.2% indicated they would recommend it to others. The distribution of KAP responses is visualized in Figure 1 (see PDF).

YES - Agreed, NO - Disagreed, CAN'T SAY - Neutral/Unsure

K- Knowledge | A- Attitude | P - Practice

Chi-square analysis revealed significant associations between KAP domains and key demographic variables: Participants aged 18-30 years showed significantly higher awareness and more favorable attitudes and practices across all items. Male respondents were significantly more likely to report awareness, ease of use and preference for telerehabilitation. Those with higher education levels demonstrated greater awareness, approachability and confidence in telerehabilitation use. A summary of statistical associations is provided in Table 2 (see PDF). Items with statistically significant differences (p < 0.05) are indicated. Telerehabilitation provides an alternative mode of delivering physiotherapy and rehabilitation services, improving access for patients beyond traditional clinical settings [1].

In this study, we examined the Knowledge, Attitude and Practice (KAP) of telerehabilitation among diverse demographic groups in Western Uttar Pradesh. By analyzing awareness, attitudes and usage patterns across age, gender and educational backgrounds, the findings provide valuable insights into public perception and the challenges surrounding the broader adoption of telerehabilitation. The limited awareness of telerehabilitation highlights a significant knowledge gap, indicating the need for targeted education campaigns [10]. Nevertheless, the strong preference for telerehabilitation indicates a positive attitude toward adopting digital healthcare technologies [11]. Ease of use was another key factor in acceptance. Many respondents reported that telerehabilitation platforms were user-friendly, fostering confidence in digital interactions for healthcare delivery [12]. However, some skepticism remained, particularly regarding whether telerehabilitation could match the effectiveness of traditional face-to-face consultations [13]. Participants also acknowledged practical advantages, with most identifying telerehabilitation as both cost- and time-efficient [14]. Furthermore, the majority considered it an accessible and convenient service. Most respondents expressed willingness to share information about telerehabilitation with others, emphasizing its potential for organic growth and broader societal acceptance [15]. Demographic analysis revealed age-related differences in perception. Younger respondents (18-30 years) demonstrated greater familiarity with technology and digital tools, making them more receptive to telerehabilitation. This age group, often referred to as digital natives, has grown up with widespread access to mobile devices and internet services, which contributes to their adaptability and comfort with virtual platforms. Consequently, they were more likely to view telerehabilitation favorably, perceiving it as an effective and efficient alternative to traditional face-to-face consultations. Their openness to digital healthcare solutions underlines the importance of leveraging technology in health promotion strategies targeting younger populations [16, 7]. While gender differences were statistically insignificant, notable trends emerged. Male respondents appeared more confident in navigating telerehabilitation platforms and were more inclined to view these services as viable, especially in emergency contexts such as sudden pain episodes. This may be attributed to greater exposure to digital tools, a higher degree of comfort with technology use and more frequent engagement with health information through online channels. Prior studies have indicated that males often report higher levels of digital literacy and self-efficacy when using health-related technologies, which could explain their favorable perception of telerehabilitation accessibility and reliability during urgent health situations [16, 17]. Educational background played a significant role in shaping KAP. Respondents with higher education levels demonstrated greater awareness, confidence and preference for telerehabilitation services. This may be attributed to their increased exposure to academic and digital environments, which foster familiarity with technology-based healthcare solutions. Higher educational attainment is often associated with better health literacy, critical thinking skills and a proactive approach to adopting new innovations, all of which contribute to the ease of use and acceptance of telerehabilitation. These findings align with earlier studies linking educational qualifications to greater digital health acceptance, reinforcing the need for targeted awareness efforts among less educated populations [12]. In summary, while the overall attitude toward telerehabilitation is positive, the study identifies crucial gaps in awareness and perceived effectiveness, especially among older adults, females and individuals with lower educational attainment. Addressing these disparities through public education, digital literacy initiatives and supportive policies will be essential to integrating telerehabilitation into mainstream healthcare systems effectively.

## Conclusion:

Telerehabilitation was widely preferred for its accessibility, cost effectiveness and usability. Younger and more educated individuals exhibited greater readiness for digital rehabilitation services. Thus, the need for targeted interventions and supportive policies to bridge demographic disparities in telerehabilitation adoption is reported.
